# Integrating spot-scanning proton arc therapy with functional avoidance strategies to reduce pulmonary toxicity

**DOI:** 10.1016/j.phro.2025.100876

**Published:** 2025-12-01

**Authors:** Yingxuan Chen, Peilin Liu, Xiaoda Cong, Edward Castillo, Richard Castillo, Inga Grills, Craig Stevens, Xiangkun Xu, Xiaoqiang Li, Yevgeniy Vinogradskiy, Xuanfeng Ding

**Affiliations:** a Department of Radiation Oncology, Thomas Jefferson University, Philadelphia, PA, United States; b Department of Radiation Oncology, Corewell Health William Beaumont University Hospital, Royal Oak, MI, United States; c Department of Biomedical Engineering, University of Texas at Austin, Austin, TX, United States; d Department of Radiation Oncology, Emory University, Atlanta, GA, United States

**Keywords:** Spot-scanning, Proton arc therapy, 4DCT-based ventilation imaging, Functional avoidance therapy, Pulmonary toxicity

## Abstract

**Background and purpose:**

Functional avoidance radiotherapy has emerged as a promising technique using functional imaging to minimize pulmonary toxicity by reducing doses to functional lung. This study aims to investigate the potential dose-volume advantages of a novel spot-scanning proton arc (SPArc) therapy for functional avoidance radiotherapy with four-dimensional computed tomography (4DCT)-based ventilation imaging.

**Material and methods:**

Twenty-five patients from a prospective functional avoidance clinical trial treated with intensity-modulated photon radiotherapy were included. Robustly optimized intensity modulated proton therapy (IMPT) and SPArc plans were generated in RayStation. Functional lung contour was derived from the 4DCT-based ventilation imaging and utilized as an optimization structure in photon as well as IMPT and SPArc functional planning. The dose distributions were compared, and normal tissue complication probability (NTCP) models were applied to estimate the probability of pulmonary toxicity.

**Results:**

Using clinical photon plans as the baseline for comparison, both proton plans achieved equivalent target coverage and reduced dose to organs at risk. Compared with photon plans, the median absolute reduction of fV_20Gy_ (the volume of functional lung receiving ≥ 20 Gy) was 3.7 percentage points (pp) with IMPT and 13.0 pp with SPArc. Using fV_20Gy_ for NTCP estimation, the median reduction of probability of grade ≥ 2 pneumonitis was 5.0 pp with IMPT and was 14.9 pp with SPArc.

**Conclusions:**

Our study highlighted the potential of SPArc to spare dose to functional lung. NTCP results further indicated that the risk of pulmonary complications can be reduced with SPArc compared to photon or IMPT for functional avoidance radiotherapy.

## Highlights

• Proton arc therapy spares functional lung dose in lung cancer radiotherapy.
• Proton arc therapy reduces mean functional lung dose by 8.2 Gy compared to photons.
• Combining functional avoidance with proton arc therapy reduces pulmonary toxicity.

## Graphical abstract

**Figure:**
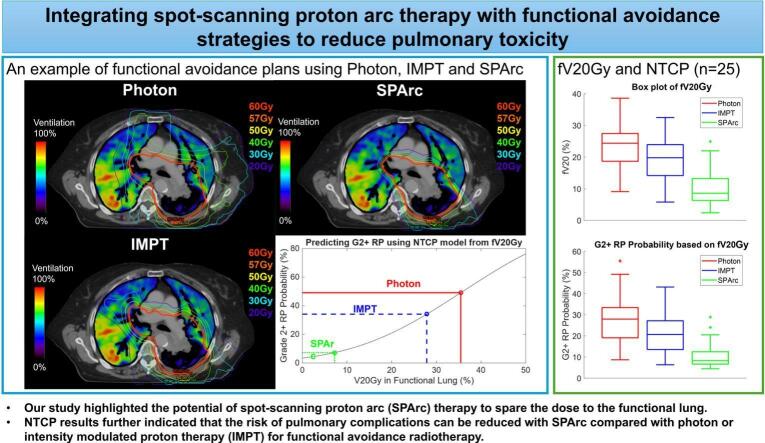

## 1 Introduction

Lung cancer patients may develop serious thoracic side effects from radiation therapy [1]. To characterize lung function, several lung functional imaging techniques have been developed that include four-dimensional computed tomography (4DCT) [2,3]. A form of lung ventilation functional imaging has been developed that uses 4DCT data along with image processing techniques to generate 4DCT-based ventilation images (referred to as ‘4DCT-ventilation’). 4DCT-ventilation is advantageous due to its wide availability and no additional cost for lung cancer patients undergoing curative radiotherapy [4,5]. The applications of 4DCT-ventilation have been demonstrated in two main areas: 1) assessing lung function and predicting treatment-related pulmonary toxicity [[6], [7], [8], [9], [10], [11]] and 2) lung functional avoidance radiotherapy [[12], [13], [14]], which aims to design treatment plans that avoid functional parts of the lung. Numerous studies have demonstrated that incorporating functional lung imaging into radiation therapy planning is a promising approach to sparing dose to functional lungs [3]. Broadly speaking, there are three main approaches: 1) a high-function region can be contoured with a pre-defined threshold and serve as an optimized structure [15] and 2) entire function imaging can be used as a factor weighting map and incorporated into a cost function [16] or 3) hybrid method by using both threshold and weighting methods [17]. The promising results of retrospective studies [3,[12], [13], [14], [15]] have prompted the initiation of numerous prospective clinical trials to further assess the feasibility and effectiveness of this approach. Recently, several clinical trials suggested a clinical benefit of embedding functional lung avoidance in the treatment planning to reduce pulmonary toxicity. A multi-institutional phase II trial (NCT02528942) demonstrated that grade ≥ 2 radiation pneumonitis (RP) can be reduced with functional avoidance radiotherapy using 4DCT-ventilation [18,19]. A single-arm prospective pilot trial (NCT02308709) confirmed significant reductions in pneumonitis incidence using this approach [20]. Additionally, a phase II single-center randomized trial (NCT02843568) proved that pulmonary toxicity can be reduced with functional lung avoidance techniques for locally advanced non-small cell lung cancer (NSCLC) patients [21].

In addition to photon therapy, proton therapy offers potential advantages for functional avoidance radiotherapy. Proton therapy has the capability to halt within a finite range in tissue and typically exhibits superior organ-at-risk (OAR) dose sparing compared to photon-based therapy. The improvement of functional lung dose sparing in the functional avoidance planning with proton therapy was investigated using double scattering as well as intensity-modulated proton therapy (IMPT), which outperformed photon-based radiotherapy contour [22,23].

Particle arc therapy has been proposed to further enhance dose distribution and sparing of healthy tissues compared to IMPT [24]. Spot-scanning Proton Arc (SPArc) therapy is an emerging technique that involves the optimization of hundreds of energy layers and thousands of spots across the arc trajectories [25]. Several *in-silico* studies have demonstrated the advantages of SPArc plans with improved target conformity and reduced OAR dose compared to IMPT for lung cancer [26]. Recently, the first prototype of spot-scanning arc treatment was developed and could be successfully delivered [27], which further highlights the clinical potential of SPArc.

There has been no work conducted to exploit the combination of SPArc with lung functional avoidance radiotherapy. Moreover, to date, functional avoidance clinical trials have focused exclusively on photon therapy [[18], [19], [20], [21]]. Thus, this study aims to quantitatively investigate the potential functional lung dose sparing and clinical benefits of combining SPArc with functional avoidance radiotherapy using 4DCT-ventilation. By comparing photon plans with two proton planning strategies (IMPT and SPArc), this work provides data-driven insights to support the integration of functionally guided proton therapy in future clinical trial designs.

## 2 Materials and methods

### 2.1 Patients

Twenty-five patients who had stage II- IV small cell lung cancer (SCLC) or NSCLC treated with definitive radiation therapy were included in this study. Patient characteristics are presented in Table 1. All patients were enrolled in a prospective functional avoidance clinical trial (NCT02528942) [19] and were treated at Corewell Health William Beaumont University Hospital (IRB# 2016–037). 4DCT imaging was acquired during the patient simulation, and the lung function map was derived from 4DCT imaging. The maximum motion of the gross tumor volume (GTV) centroid was reported in the assessment of motion.

**Table 1:** Patient characteristics. LUL = left upper lobe; LLL = left lower lobe; RUL = right upper lobe; RML = right middle lobe; RLL = right lower lobe; CTV = clinical target volume on the average CT images.

Characteristics	No. (%) or Median (range)
Patient no.	25
Gender	
Male	13 (52 %)
Female	12 (48 %)
Age, years	69 (54–84)
Smoking Status	
Nonsmoker	1 (4 %)
Current Smoker	5 (20 %)
Former Smoker	19 (76 %)
Diagnosis	
SCLC	1 (4 %)
NSCLC	24 (96 %)
Tumor location	
LUL	6 (24 %)
LLL	3 (12 %)
RUL	5 (20 %)
RML	6 (24 %)
RLL	5 (20 %)
Stage	
I	0 (0 %)
II	2 (8 %)
III	22 (88 %)
IV	1 (4 %)
Chemotherapy	
Concurrent	23 (92 %)
Sequential	2 (8 %)
Immunotherapy	
Yes	9 (36 %)
No	16 (64 %)
Radiation prescription	
Total dose (Gy)	60 (45––60)
Number of fractions	30 (25––30)
Fractionation pattern	
Daily	24 (96 %)
Twice daily	1 (4 %)
CTV volume (cm^3^)	268 (88–834)
Max. tumor motion (cm)	0.5 (0.2–1.1)

### 2.2 Ventilation imaging and high functional lung contour generation

4DCT images were used to generate lung ventilation imaging using CT number density-change-based method [28,29]. The inhale phase images were mapped to the exhale phase images via deformable image registration [30,31]. Then, ventilation within the lungs was calculated based on the density change using Equation (1) [4,32]:

(1) $$\frac{V_{\mathrm{in}}-V_{\mathrm{ex}}}{V_{\mathrm{ex}}}=1000\frac{f_{\mathrm{in}}-f_{\mathrm{ex}}}{f_{\mathrm{ex}}\left(1000+f_{\mathrm{in}}\right)}$$

where *V_in_* and *V_ex_* represent the volumes of air within the inhale and exhale CT voxel pair, respectively. *f*_ex_ and *f*_in_ are the CT numbers of the individual lung voxels of the exhale CT and the deformed inhale CT, respectively.

The functional lung contour was then auto-segmented using a pre-defined threshold on 4DCT-ventilation. This threshold was determined through the area under the curve (AUC) analysis to identify the optimal operating point. Based on the previous studies, the functional lung contour includes lung regions with at least a 15 % reduction in the mean value of ventilation [33].

### 2.3 4DCT-based functional avoidance planning

In this study, an internal target volume (ITV) based approach was used. Physicians contoured GTVs on 10 phases to generate the GTV-ITV and subsequently defined the clinical target volume (CTV) by expanding the GTV-ITV with a 5 mm margin. The average CT was utilized for all planning strategies. In addition, a structure-based functional avoidance planning strategy was applied in photon radiotherapy (using intensity modulated radiation therapy (IMRT) or volumetric-modulated arc therapy (VMAT)) as well as IMPT and SPArc.

In clinical functional photon planning, the planning target volume (PTV) was created by adding a 5 mm uniform margin to the CTV. The clinical photon plan was generated with the Pinnacle (Philips Healthcare) treatment planning system using an Elekta Agility (Elekta, Stockholm, Sweden) following instruction guidelines from the Radiation Therapy Oncology group (RTOG) 0617 and RTOG 0538 (for SCLC). Multi-field static IMRT or the VMAT planning technique was used for functional avoidance planning for photon radiotherapy. The treatment planners were instructed not to sacrifice target coverage and standard OAR constraints when reducing the dose to the functional lung contour. To efficiently optimize the dose, a functional avoid optimization structure was generated using this functional lung contour with subsequent subtraction from PTV with a 5 mm margin and used as an optimization structure. The photon plans were approved by radiation oncologists and used to treat patients enrolled in the prospective clinical trial.

In this study, both IMPT and SPArc plans were generated retrospectively in the RayStation (RaySearch Laboratories, Stockholm, Sweden) planning system. To account for the biological effectiveness of protons, a constant factor of 1.1 was considered [34]. The worst-case scenario robust optimization was applied to the CTV for the target coverage against 21 scenarios (including 5 mm setup and 3.5 % range uncertainties) for both proton plans (IMPT and SPArc). During the optimization, the planners were instructed to reduce the dose to the functional lung while ensuring adequate target volume coverage and compliance with dose constraints for OARs. Note that the same functional avoid optimization structure was used for proton optimization. IMPT plans were generated using the single-field optimization (SFO) approach in the RayStation [26], which reflects the current clinical standard at our institution. Specifically, two or three fields were selected based on the target and functional lung structure location. The SPArc plan was generated using an in-house developed algorithm implemented in RayStation [25]. Unlike IMPT, which used two or three fields, a partial arc trajectory with a sampling frequency of 2.5 degrees was used in SPArc planning.

### 2.4 Plan evaluation

The dose volume histograms (DVHs) for CTV, GTV-ITV, and OARs were calculated for plan evaluation. For proton plans, DVHs in the nominal scenario were extracted to compare with photon plans. Functional lung dose was evaluated using fV_5Gy_, fV_10Gy_, fV_20Gy_, fV_30Gy_ (relative volumes receiving ≥ 5, 10, 20, and 30 Gy) and the mean functional lung dose (fMLD). To further assess dose-volume parameters differences between photon and proton plans, these metrics were also reported separately for the ipsilateral and contralateral functional lungs. The clinical functional photon plans were used as the baseline for comparison. The absolute reductions in dose-volume parameters achieved with IMPT or SPArc were reported in terms of median values and ranges. In addition, the Wilcoxon signed rank test was performed to evaluate differences between functional photon plans and functional proton plans (IMPT or SPArc).

In order to evaluate how the dose-volume differences translated to clinical outcomes, the functional lung dose metrics were used to calculate pneumonitis normal tissue complication probabilities (NTCP) based on previous published functional lung NTCP models [15]. Using these NTCP models, the probabilities of grade ≥ 2 and grade ≥ 3 RP were predicted based on functional lung dose metrics, such as fV_20Gy_ or fMLD. Note that the same functional lung contour was used for both the dose and NTCP evaluation.

The delivery time for photon radiotherapy was recorded in the Oncology Information System (OIS). A published Dynamic Arc system simulator was used in this study to estimate the total treatment delivery time for IMPT and SPArc plans. The simulator was validated using a clinical IBA ProteusPLUS® in which the continuous proton irradiation is enabled during the dynamic gantry rotation [35]. The mechanical limitation of the proton gantry system was considered with the maximum rotation speed of 6 deg/s and the maximum acceleration or deceleration speed of 0.6 deg/s^2^. For the IMPT treatment delivery simulation, the tolerance window was set at 0°, which indicates that the gantry requires a full stop before delivery of the proton beam. The delivery time for the photon, IMPT, and SPArc were compared for the treatment efficiency evaluation.

## 3 Results

### 3.1 Plan comparison

**Fig. 1:**
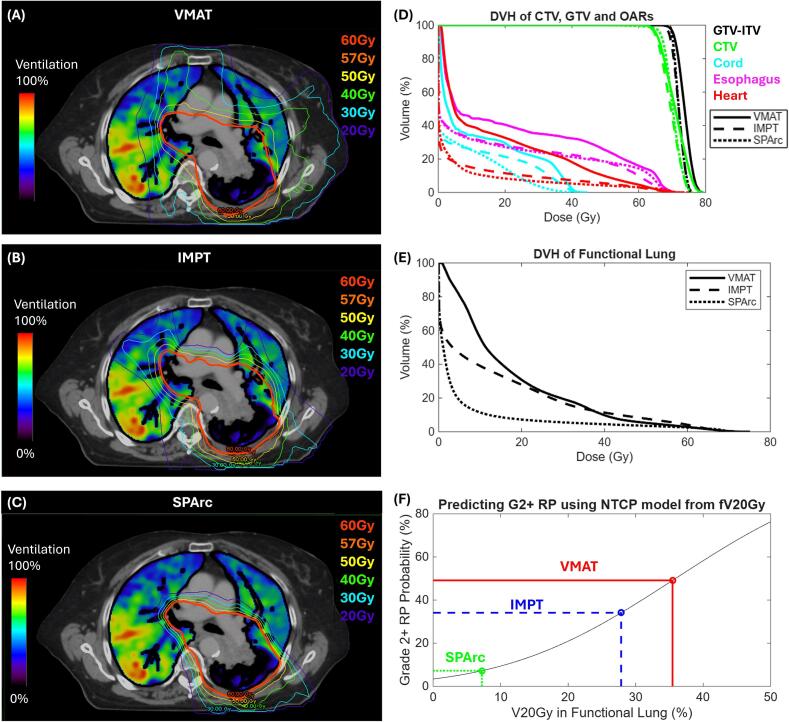
Functional plans of an example patient are shown for (A) VMAT (clinical treated), (B) two-field IMPT (beam angles are 120° and 180°) and (C) SPArc. (D) DVH comparison between VMAT, IMPT and SPArc for CTV, GTV-ITV and OARs. (E) DVH of the functional lung comparison between three planning techniques. (F) NTCP model for grade ≥ 2 RP as a function of fV_20Gy_.

Fig. 1 (A-E) shows an example patient with three plans. Compared to VMAT, IMPT reduced the dose to the functional lung, while SPArc achieved an even larger dose reduction around the CTV. For this representative patient, fV_20Gy_ was 35.5 % with VMAT, which decreased to 27.9 % using IMPT, and was further reduced to 7.2 % with SPArc.

Table 2 presents a detailed dose-volume parameters comparison among photon, IMPT, and SPArc plans across all 25 patients. The average CTV D_99%_ values were 61.14 Gy, 61.47 Gy, and 61.05 Gy for the VMAT, IMPT, and SPArc plans, respectively. Moreover, robustness analysis of IMPT and SPArc plans showed that in all 21 scenarios, CTV D_95%_ remained above 95 % of the prescription dose. Compared with photon plans, doses to OARs were significantly reduced with both proton plans (p < 0.001).

**Table 2:** Dose-volume comparisons for functional avoidance plans using different planning techniques: intensity modulated photon therapy, IMPT and SPArc (n = 25). Wilcoxon signed rank test P value was performed between photon plans and proton plans (IMPT or SPArc).

	**Photon**	**IMPT**	**Photon vs. IMPT**	**SPArc**	**Photon vs. SPArc**
	Mean ± SD	Mean ± SD	Wilcoxon signed rank test P value	Mean ± SD	Wilcoxon signed rank test P value
**CTV D_0.03cm3_ (Gy)**	75.1 ± 7.4	73.7 ± 7.2	0.03	73.2 ± 6.7	<0.01
**CTV D_99%_ (Gy)**	61.1 ± 5.4	61.5 ± 6.1	0.24	61.1 ± 5.6	0.78
**GTV-ITV D_99%_ (Gy)**	65.6 ± 7.0	65.3 ± 7.2	0.07	65.5 ± 7.2	0.57
**Esophagus mean (Gy)**	23.6 ± 9.7	17.6 ± 12.3	<0.01	13.8 ± 10.6	<0.01
**Heart mean (Gy)**	13.1 ± 6.9	5.2 ± 3.2	<0.01	4.4 ± 3.3	<0.01
**Cord Max (Gy)**	36.6 ± 8.7	26.6 ± 13.1	<0.01	24.4 ± 13.0	<0.01
**Total lung V_20Gy_ (%)**	26.0 ± 5.8	21.5 ± 5.4	<0.01	15.3 ± 5.5	<0.01
**Total lung Mean (Gy)**	15.6 ± 3.0	11.0 ± 2.8	<0.01	8.6 ± 2.9	<0.01
**Functional lung metrics**					
**Ipsilateral functional lung**					
**fMLD (Gy)**	24.5 ± 6.6	20.9 ± 6.9	<0.01	14.3 ± 6.6	<0.01
**fV_5Gy_ (%)**	78.3 ± 17.2	62.3 ± 19.9	<0.01	47.4 ± 19.2	<0.01
**fV_10Gy_ (%)**	67.5 ± 18.2	56.0 ± 20.0	<0.01	35.4 ± 17.5	<0.01
**fV_20Gy_ (%)**	50.2 ± 16.1	42.5 ± 15.8	<0.01	24.6 ± 14.2	<0.01
**fV_30Gy_ (%)**	35.6 ± 14.0	30.3 ± 12.0	<0.01	18.6 ± 10.9	<0.01
**fV_40Gy_ (%)**	24.5 ± 10.6	21.6 ± 8.8	0.01	14.5 ± 8.7	<0.01
**fV_50Gy_ (%)**	14.6 ± 7.5	14.9 ± 6.7	0.16	10.8 ± 6.9	<0.01
**Contralateral functional lung**					
**fMLD (Gy)**	7.4 ± 2.7	1.2 ± 2.1	<0.01	0.7 ± 1.1	<0.01
**fV_5Gy_ (%)**	60.7 ± 16.3	6.3 ± 11.5	<0.01	3.1 ± 6.6	<0.01
**fV_10Gy_ (%)**	21.7 ± 17.8	3.7 ± 7.9	<0.01	1.2 ± 2.7	<0.01
**fV_20Gy_ (%)**	3.3 ± 5.8	1.5 ± 3.7	<0.01	0.4 ± 0.9	<0.01
**fV_30Gy_ (%)**	1.2 ± 2.7	0.7 ± 1.6	<0.01	0.2 ± 0.4	<0.01
**fV_40Gy_ (%)**	0.4 ± 1.2	0.3 ± 0.7	0.01	0.1 ± 0.2	<0.01
**fV_50Gy_ (%)**	0.1 ± 0.3	0.10 ± 0.3	0.25	0.1 ± 0.1	0.23
**Total functional lung**					
**fMLD (Gy)**	14.6 ± 3.6	9.5 ± 3.6	<0.01	6.5 ± 3.2	<0.01
**fV_5Gy_ (%)**	68.3 ± 13.7	30.2 ± 13.2	<0.01	21.8 ± 10.9	<0.01
**fV_10Gy_ (%)**	41.3 ± 13.0	25.9 ± 11.5	<0.01	15.5 ± 7.8	<0.01
**fV_20Gy_ (%)**	23.0 ± 7.9	18.7 ± 7.3	<0.01	10.5 ± 5.9	<0.01
**fV_30Gy_ (%)**	15.5 ± 6.3	13.0 ± 5.2	<0.01	8.0 ± 5.0	<0.01
**fV_40Gy_ (%)**	10.6 ± 5.3	9.2 ± 4.1	<0.01	6.2 ± 4.2	<0.01
**fV_50Gy_ (%)**	6.3 ± 3.7	6.4 ± 3.3	0.17	4.7 ± 3.5	<0.01

**Fig. 2:**
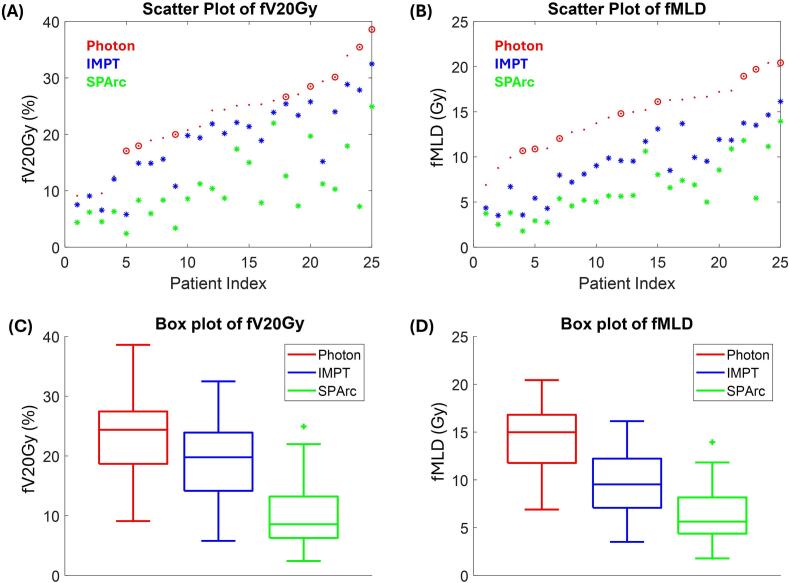
Dose to functional lung was compared using three different treatment techniques: photon, IMPT, and SPArc. (A) and (B) show a scatter plot of fV_20Gy_ and fMLD for all 25 patients. 8 patients with G2 + RP were highlighted with circles. (C) and (D) are box plots of fV_20Gy_ and fMLD from functional photon plan, IMPT, and SPArc.

Fig. 2 shows that both proton plans reduced dose to functional lung contour. Compared to photon therapy, IMPT reduced fV_20Gy_ by a median of 3.7 percentage points (pp), ranging from 0.1 to 14.2 pp, while SPArc achieved a larger absolute reduction with a median of 13.0 pp (range: 3.0 to 28.3 pp). fMLD was reduced by a median of 5.3  Gy (range: 2.6–7.8  Gy) with IMPT and 8.2  Gy (range: 3.2–14.3  Gy) with SPArc. Additional details on the correlation between functional lung dose and the incidence of RP are provided in Supplementary A.

### 3.2 Normal tissue complication probability (NTCP) comparison

Fig. 1(F) illustrates the NTCP model for predicting for grade ≥ 2 RP, with fV_20Gy_ as the dose metric input for the example patient. NTCP value was 34.1 % with IMPT, 7.2 % using SPArc, compared to 49.1 % with the clinical photon plan.

**Fig. 3:**
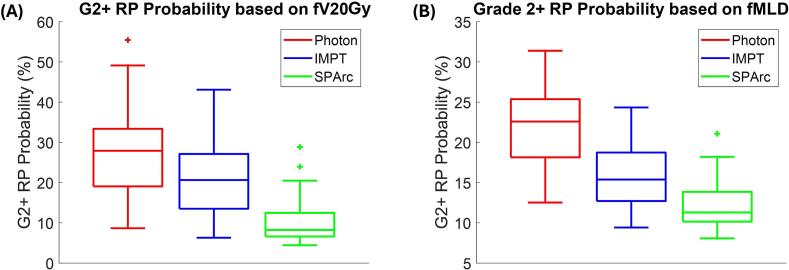
(A) and (B) show that grade ≥ 2 RP probabilities using functional lung fV_20Gy_ or fMLD can be reduced using IMPT and can be further reduced using SPArc compared with the intensity modulated photon treatment.

Fig. 3 presents box plots of the NTCP values for grade ≥ 2 RP prediction using models based on fV_20Gy_ and fMLD across photon, IMPT, and SPArc plans. Using fV_20Gy_ for NTCP estimation, IMPT significantly reduced the probability of grade ≥ 2 RP compared to photon therapy, with a median absolute reduction of 5.0 pp (range: 0.1 to 22.4 pp) (p < 0.001). SPArc achieved a significantly greater reduction, with a median of 14.9 pp (range: 2.2 to 41.9 pp) (p < 0.001). NTCP for G2 + RP prediction was also calculated using the models based on other dose metrics (including fV_5Gy_, fV_10Gy_, fV_30Gy_, or fMLD), as shown in Supplemental Table S1. Furthermore, NTCP values for G3 + RP are presented in Supplemental Table S2.

### 3.3 Delivery time comparison

Six out of 25 patients were treated using multi-field static IMRT, and the total delivery time was (mean ± standard deviation) 20.5 ± 7.7 min, including machine mod-up time for each treatment field. The remaining 19 patients were treated using VMAT with an average delivery time of 3.3 ± 1.0 min. For the IMPT plans, 2 or 3 proton fields were used in the functional avoidance plan and estimated delivery time is 3.5 ± 1.3 min over all 25 patients. The estimated delivery time for SPArc was 3.7 ± 1.0 min, which was comparable to the delivery time of VMAT.

## 4 Discussion

This study demonstrated the potential of SPArc to improve functional lung dose sparing in 4DCT-derived lung functional avoidance planning. Compared to conventional photon-based approaches, SPArc achieved superior sparing of functional lung tissue without compromising target coverage. SPArc consistently reduced functional lung dose, resulting in a reduced risk of radiation-induced pulmonary complications predicted by NTCP modeling, underscoring its promise for functional avoidance radiotherapy in lung cancer treatment.

In this study, functional IMPT reduced the fMLD by 5.1 Gy on average and fV_20Gy_ by 4.3 pp compared to the photon plans. The average reduction of fMLD and fV_20Gy_ was reported to be 7.2 Gy and 9.2 pp in the study by Dougherty et al. [23]. The differences in functional dose reductions between the current work and the study by Dougherty et al. can be attributed to that multi-field optimization (MFO) was used in their study while single-field optimization (SFO) was used in our study. Compared to MFO, SFO proton planning is expected to provide relatively limited reductions in functional lung dose [36]. Although MFO can offer larger dose sparing, we opted to use SFO for IMPT planning to align with our institution’s clinical protocol to ensure a clinically meaningful comparison. SPArc reduced functional lung dose with an average of 3.1 Gy on fMLD and 8.2 pp on fV_20Gy_ compared to IMPT. Additional details on the dose–volume comparison between IMPT and SPArc are provided in Supplementary Table S3.

Moreover, as shown in Fig. 2 (A and B), variabilities in the reduction of functional lung dose were observed among the 25 patients. In the future, further investigations will be conducted to identify which patients would benefit from proton treatments, particularly those who could gain from SPArc in managing pulmonary complication risk and aiding clinical decision-making.

This *in-silico* study highlights an important principle in radiation oncology: the enhanced flexibility and increased degree of freedom in optimization and treatment delivery can significantly improve dose-volume plan quality. The increased flexibility is especially valuable for clinical cases involving complex geometries or structures, such as those presented with functional avoidance radiotherapy. Such a principle applies not only to photon radiotherapy, e.g., Cyberknife [37], Gamma Knife [38], and HyperArc [39], but also to particle beam therapies, where we see a dose improvement moving from static field treatment to dynamic arc therapy [25,40]. As radiotherapy progresses toward personalized treatment, functional imaging techniques enable the extraction of patient-specific data, further refining treatment plans for improved outcomes, which normally generates a complicated voxel-based structure for OARs dose sparing or target dose escalation [[41], [42], [43]].

Compared to photon therapy, proton therapy is more sensitive to uncertainties in dose delivery induced by respiratory motion. To minimize the impact of motion on dose delivery, several motion management strategies have been recommended in the American Association of Physicists in Medicine Task Group 290 [44]. Previous studies have demonstrated that functional IMPT plans exhibit robustness against the motion interplay effect [23]. Furthermore, SPArc has shown superior robustness compared to conventional IMPT in the presence of tumor motion [26,45]. An ITV-based approach was employed in this study to account for tumor motion. Future work may incorporate advanced motion management techniques, such as direct 4D dose optimization [46], in conjunction with functional lung avoidance strategies to further enhance the robustness of proton plans.

One limitation of our study was that the NTCP models for RP prediction were fitted based on functional lung dose from photon plans. The accuracy of the NTCP models might be limited for the proton treatment. Several studies have already shown that the dose metrics to predict RP might be different between photon and proton treatments for lung cancer patients [47,48]. O’Reilly et al demonstrated that the high functional lung dose can better predict the incidence of RP compared to the traditional dose-volume metrics for NSCLC patients treated with photon or proton. In their study, the mean relative volume of the high-ventilation lung contour receiving > 20 Gy in the pneumonitis group were 20.6 % and 18.4 % for photon and proton treatments, respectively [49]. The functional lung dose constraints for proton to prevent RP might be different than for photon. Further studies on the development of specific NTCP models for proton treatments could be of interest.

Another limitation of this study is the limited patient cohort, with 25 patients included in the analysis. Future research with larger, more diverse patient cohorts is essential to validate these findings, strengthen statistical confidence, and provide a more comprehensive understanding of functional lung avoidance therapy using different planning strategies.

This study, which optimized the dose to spare lung ventilation function, marked the initial stage to investigate the feasibility of functional and biological-guided particle therapy using SPArc technology. Furthermore, significant tumor reduction and airway re-opening have been demonstrated throughout the course of treatment using weekly 4DCT-ventilation [50]. In cases where significant lung function changes occur throughout treatment, adapting the treatment plan based on the mid-treatment ventilation maps can add great clinical benefit [51]. Future clinical exploration would extend to functional adaptive therapy throughout treatment with the guidance of functional imaging such as 4DCT-ventilation.

In summary, this was the first work that demonstrated the potential of SPArc to spare dose to the functional lung while maintaining satisfying target coverage. Compared to photon plans, fV_20Gy_ was reduced by a median of 13.0 pp, and fMLD was reduced by a median of 8.2 Gy with SPArc. Furthermore, NTCP results indicate that the risk of pulmonary complications can be reduced with SPArc for functional avoidance radiotherapy.

## Declaration of competing interest

The authors declare the following financial interests/personal relationships which may be considered as potential competing interests: Xuanfeng Ding holds a patent related to Particle Arc Therapy, and it has been assigned to Corewell Health and licensed to IBA. Xuanfeng Ding received honoraria and research funding from IBA and Elekta Speaker Bureau outside the work presented here.

Other authors have no known competing financial interests or personal relationships.
