# Supplementary material for: Integrating spot-scanning proton arc therapy with functional avoidance strategies to reduce pulmonary toxicity
Source: Phys Imaging Radiat Oncol. 2025 Dec 1;36:100876. doi: 10.1016/j.phro.2025.100876 (PMC12721199; doi:10.1016/j.phro.2025.100876)
Supplement: Supplementary Data 1 [file mmc1.pdf]

Supplementary

Table S1. Normal tissue complication probability (NTCP) values for G2+ radiation pneumonitis were shown here in the format of the median (range) using the NTCP models based on functional lung dose metrics (fV_{5Gy}, fV_{10Gy}, fV_{20Gy}, fV_{30Gy}, and mean functional dose fMLD)

**G2+ toxicity**			
**Photon vs. IMPT**			
Dose Metric for NTCP	Photon (%)	IMPT (%)	Absolute reduction (percentage points)
fV_{5Gy}	46.6 (14.0 to 69.3)	10.5 (3.6 to 43.3)	32.8 (9.1 to 50.2)
fV_{10Gy}	31.9 (10.1 to 68.4)	16.4 (5.6 to 51.3)	15.4 (1.5 to 41.7)
fV_{20Gy}	27.9 (8.7 to 55.4)	20.6 (6.3 to 43.1)	5.0 (0.1 to 22.4)
fV_{30Gy}	23.6 (10.3 to 46.6)	19.0 (9.6 to 37.1)	3.2 (-0.7 to 13.6)
fMLD	22.6 (12.5 to 31.4)	15.4 (9.4 to 24.3)	6.9 (2.4 to 10.4)
**Photon vs. SPArc**			
Dose Metric for NTCP	Photon (%)	SPArc (%)	Absolute reduction (percentage points)
fV_{5Gy}	46.6 (14.0 to 69.3)	6.6 (2.9 to 25.0)	37.3 (8.7 to 56.2)
fV_{10Gy}	31.9 (10.1 to 68.4)	7.9 (4.0 to 25.6)	19.8 (3.1 to 61.5)
fV_{20Gy}	27.9 (8.7 to 55.4)	8.3 (4.4 to 28.9)	14.9 (2.2 to 41.9)
fV_{30Gy}	23.6 (10.3 to 46.6)	11.9 (8.0 to 30.3)	10.2 (0.8 to 23.2)
fMLD	22.6 (12.5 to 31.4)	11.3 (8.1 to 21.1)	10.3 (2.9 to 19.1)
**IMPT vs. SPArc**			
Dose Metric for NTCP	IMPT (%)	SPArc (%)	Absolute reduction (percentage points)
fV_{5Gy}	10.5 (3.6 to 43.3)	6.6 (2.9 to 25.0)	3.6 (-0.3 to 18.3)
fV_{10Gy}	16.4 (5.6 to 51.3)	7.9 (4.0 to 25.6)	8.1 (0.2 to 37.3)
fV_{20Gy}	20.6 (6.3 to 43.1)	8.3 (4.4 to 28.9)	8.6 (1.3 to 26.9)
fV_{30Gy}	19.0 (9.6 to 37.1)	11.9 (8.0 to 30.3)	6.0 (1.5 to 14.8)
fMLD	15.4 (9.4 to 24.3)	11.3 (8.1 to 21.1)	2.9 (0.5 to 9.4)

Table S2. Normal tissue complication probability (NTCP) values for G3+ radiation pneumonitis were shown here in the format of the median (range) using the NTCP models based on functional lung dose metrics (fV5Gy, fV10Gy, fV20Gy, fV30Gy, and mean functional dose fMLD)

**G3+ toxicity**			
**Photon vs. IMPT**			
Dose Metric for NTCP	Photon (%)	IMPT (%)	Absolute reduction (percentage points)
fV_{5Gy}	20.6 (18.4 to 22.0)	18.1 (16.9 to 20.4)	2.4 (1.2 to 3.9)
fV_{10Gy}	18.5 (11.3 to 29.9)	13.7 (9.1 to 24.2)	4.9 (0.5 to 12.7)
fV_{20Gy}	13.6 (4.1 to 30.6)	9.8 (3.1 to 22.3)	2.6 (0.1 to 11.7)
fV_{30Gy}	10.8 (4.2 to 25.3)	8.4 (3.9 to 18.8)	1.8 (-0.3 to 8.5)
fMLD	10.3 (5.2 to 15.2)	6.6 (3.8 to 11.2)	3.3 (1.1 to 5.4)
**Photon vs. SPArc**			
Dose Metric for NTCP	Photon (%)	SPArc (%)	Absolute reduction (percentage points)
fV_{5Gy}	20.6 (18.4 to 22.0)	17.5 (16.7 to 19.3)	2.7 (1.1 to 4.1)
fV_{10Gy}	18.5 (11.3 to 29.9)	10.3 (8.1 to 19.6)	7.0 (1.4 to 20.2)
fV_{20Gy}	13.6 (4.1 to 30.6)	4.0 (2.2 to 14.1)	7.5 (1.0 to 22.7)
fV_{30Gy}	10.8 (4.2 to 25.3)	4.9 (3.2 to 14.6)	5.5 (0.4 to 12.5)
fMLD	10.3 (5.2 to 15.2)	4.7 (3.2 to 9.5)	5.1 (1.3 to 9.9)
**IMPT vs. SPArc**			
Dose Metric for NTCP	IMPT (%)	SPArc (%)	Absolute reduction (percentage points)
fV_{5Gy}	18.1 (16.9 to 20.4)	17.5 (16.7 to 19.3)	0.4 (-0.1 to 1.7)
fV_{10Gy}	13.7 (9.1 to 24.2)	10.3 (8.1 to 19.6)	3.0 (0.1 to 11.4)
fV_{20Gy}	9.8 (3.1 to 22.3)	4.0 (2.2 to 14.1)	4.2 (0.6 to 13.5)
fV_{30Gy}	8.4 (3.9 to 18.8)	4.9 (3.2 to 14.6)	3.0 (0.7 to 7.5)
fMLD	6.6 (3.8 to 11.2)	4.7 (3.2 to 9.5)	1.3 (0.2 to 4.6)

Table S3. Dosimetric results comparisons for functional avoidance proton plans using different planning techniques: IMPT and SPArc. (n=25)

	IMPT	SPArc	
	Mean± SD	Mean± SD	Wilcoxon signed rank test P value
**CTV 0.03cm^3 (Gy)**	73.70 ±7.19	73.19 ± 6.70	0.819
**CTV D99% (Gy)**	61.47 ± 6.06	61.05 ± 5.63	0.115
**GTV_ITV D99% (Gy)**	65.26 ± 7.16	65.45 ± 7.22	0.346
**Esophagus mean (Gy)**	17.60 ± 12.27	13.79 ± 10.57	<.001
**Heart mean (Gy)**	5.15 ± 3.16	4.36 ± 3.27	<.001
**Cord Max (Gy)**	26.61 ± 13.11	24.43 ± 12.98	0.226
**Total lung V20 (%)**	21.46 ± 5.43	15.28 ± 5.51	<.001
**Total lung Mean (Gy)**	11.03 ± 2.83	8.64 ± 2.86	<.001
**Functional lung metrics**			
**Ipsilateral functional lung**			
**fMLD (Gy)**	20.90 ± 6.93	14.32 ± 6.57	<.001
**fV_{5Gy} (%)**	62.34 ± 19.86	47.39 ± 19.19	<.001
**fV_{10Gy} (%)**	56.04 ± 19.99	35.39 ± 17.45	<.001
**fV_{20Gy} (%)**	42.48 ± 15.82	24.58 ± 14.18	<.001
**fV_{30Gy} (%)**	30.26 ± 11.98	18.64 ± 10.93	<.001
**fV_{40Gy} (%)**	21.55 ± 8.77	14.48 ± 8.73	<.001
**fV_{50Gy} (%)**	14.89 ± 6.67	10.76 ± 6.88	<.001
**Contralateral functional lung**			
**fMLD (Gy)**	1.17 ± 2.10	0.68 ± 1.05	0.008
**fV_{5Gy} (%)**	6.33 ± 11.46	3.13 ± 6.59	0.001
**fV_{10Gy} (%)**	3.71 ±7.88	1.19 ± 2.72	<.001
**fV_{20Gy} (%)**	1.51 ± 3.74	0.42 ± 0.87	<.001
**fV_{30Gy} (%)**	0.65 ± 1.61	0.20 ± 0.38	0.016
**fV_{40Gy} (%)**	0.26 ± 0.67	0.10 ± 0.19	0.078
**fV_{50Gy} (%)**	0.10 ± 0.30	0.05 ± 0.11	0.375
**Total functional lung**			
**fMLD (Gy)**	9.50 ± 3.60	6.45 ± 3.18	<.001
**fV_{5Gy} (%)**	30.18 ± 13.19	21.80 ± 10.86	<.001
**fV_{10Gy} (%)**	25.91 ± 11.50	15.48 ± 7.76	<.001
**fV_{20Gy} (%)**	18.70 ± 7.27	10.49 ± 5.93	<.001
**fV_{30Gy} (%)**	12.97 ± 5.17	7.97 ± 4.97	<.001
**fV_{40Gy} (%)**	9.18 ± 4.12	6.22 ± 4.23	<.001
**fV_{50Gy} (%)**	6.35 ± 3.31	4.67 ± 3.48	<.001

Supplementary A

Correlation of functional lung dose with incidence of RP

Eight out of 25 patients developed grade 2 or higher radiation pneumonitis. The dose difference between the two groups (G2+ pneumonitis vs. G0 or G1 pneumonitis) was compared for each planning technique. An unpaired t-test was performed between the two groups.

Boxplots of the differences in dosimetric parameters fV_{20Gy} or fMLD for two groups (G2+ pneumonitis vs. G0 or G1 pneumonitis) are shown in Figure S1. The incidence of RP was based on photon plans. A non-paired T-test was performed, and *p* values show that there were no significant differences between these two patient groups regarding fV_{20Gy} or fMLD. Both proton planning techniques can reduce the dose to the functional lung for both groups. The difference between dosimetric parameters shows a non-significant difference between the two groups in the *in-silico* proton plans with increasing *p* values.

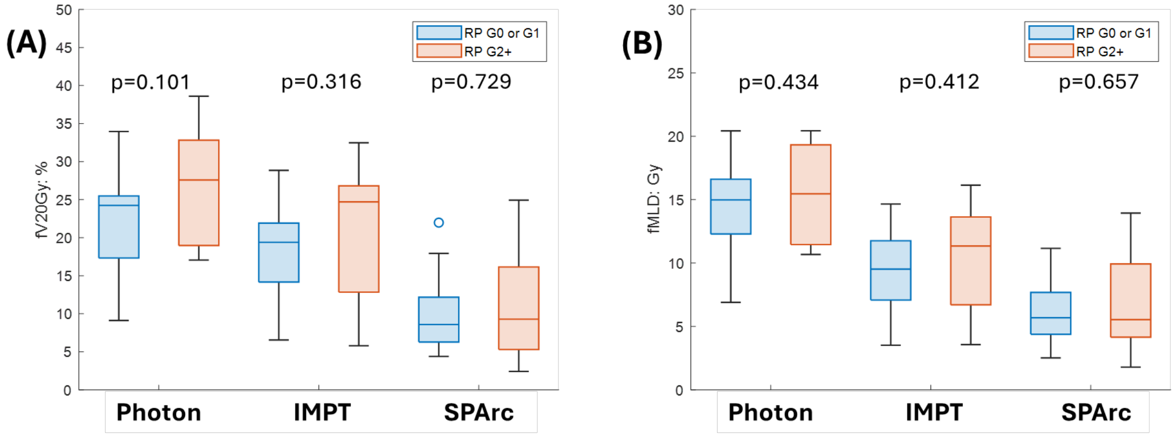

Figure S1. (A) and (B) are box plots of the differences in dosimetric parameters fV_{20Gy} or fMLD for two groups (G2+ pneumonitis vs. G0 or G1 pneumonitis).
